# Moderate positive predictive value of a multiplex real-time PCR on whole blood for pathogen detection in critically ill patients with sepsis

**DOI:** 10.1007/s10096-019-03616-w

**Published:** 2019-06-26

**Authors:** Kirsten van de Groep, Martine P. Bos, Meri R. J. Varkila, Paul H. M. Savelkoul, David S. Y. Ong, Lennie P. G. Derde, Nicole P. Juffermans, Tom van der Poll, Marc J. M. Bonten, Olaf L. Cremer, on behalf of the MARS consortium

**Affiliations:** 1 Julius Center for Health Sciences and Primary Care, University Medical Center Utrecht, Utrecht University, Utrecht, The Netherlands; 2 Department of Intensive Care Medicine, University Medical Center Utrecht, Utrecht University, Utrecht, The Netherlands; 3 Microbiome, Amsterdam, The Netherlands; 4 Department of Medical Microbiology & Infection Control, Amsterdam University Medical Centers, VU, Amsterdam, The Netherlands; 5 Department of Medical Microbiology, Maastricht University Medical Center, Maastricht, The Netherlands; 6 Department of Medical Microbiology and Infection Control, Franciscus Gasthuis & Vlietland, Rotterdam, The Netherlands; 7 Department of Intensive Care, Amsterdam University Medical Centers, Academic Medical Center, University of Amsterdam, Amsterdam, The Netherlands; 8 Center of Experimental and Molecular Medicine, Amsterdam University Medical Centers, Academic Medical Center, University of Amsterdam, Amsterdam, The Netherlands; 9 Division of Infectious Diseases, Amsterdam University Medical Centers, Academic Medical Center, University of Amsterdam, Amsterdam, The Netherlands; 10 Department of Medical Microbiology, University Medical Center Utrecht,, Utrecht University, Utrecht, The Netherlands

**Keywords:** Diagnostic research, Pathogen detection, Sepsis, Intensive care, Multiplex real-time PCR

## Abstract

A novel multiplex real-time PCR for bloodstream infections (BSI-PCR) detects pathogens directly in blood. This study aimed at determining the positive predictive value (PPV) of BSI-PCR in critically ill patients with sepsis. We included consecutive patients with presumed sepsis upon admission to the intensive care unit (ICU). The multiplexed BSI-PCR included 17 individual PCRs for a broad panel of species- and genus-specific DNA targets. BSI-PCR results were compared with a reference diagnosis for which plausibility of infection and causative pathogen(s) had been prospectively assessed by trained observers, based on available clinical and microbiological evidence. PPV and false positive proportion (FPP) were calculated. Clinical plausibility of discordant positive results was adjudicated by an expert panel. Among 325 patients, infection likelihood was categorized as confirmed, uncertain, and ruled out in 210 (65%), 88 (27%), and 27 (8%) subjects, respectively. BSI-PCR identified one or more microorganisms in 169 (52%) patients, of whom 104 (61%) had at least one detection in accordance with the reference diagnosis. Discordant positive PCR results were observed in 95 patients, including 30 subjects categorized as having an “unknown” pathogen. Based on 5525 individual PCRs yielding 295 positive results, PPV was 167/295 (57%) and FPP was 128/5525 (2%). Expert adjudication of the 128 discordant PCR findings resulted in an adjusted PPV of 68% and FPP of 2%. BSI-PCR was all-negative in 156 patients, including 79 (51%) patients in whom infection was considered ruled out. BSI-PCR may complement conventional cultures and expedite the microbiological diagnosis of sepsis in ICU patients, but improvements in positive predictive value of the test are warranted before its implementation in clinical practice can be considered.

**Electronic supplementary material:**

The online version of this article (10.1007/s10096-019-03616-w) contains supplementary material, which is available to authorized users.

## Introduction

Diagnosing sepsis can be challenging in critically ill patients having multiple concurrent disease processes. Collection of microbiological evidence for infection is therefore of importance to establish the diagnosis and optimize antimicrobial treatment [1]. However, sensitivity of culture-based methods is suboptimal and previous antibiotic exposure may render results unreliable [2–4]. Moreover, turnaround times for culture-based methods (if used in combination with MALDI-TOF MS identification) range from 12 to 72 h from sampling until final result [5]. As a result, initiation of antimicrobial treatment in the intensive care unit (ICU) is mostly empirical [6].

Several molecular approaches have been developed in an attempt to improve the diagnosis of sepsis—and bloodstream infections (BSI) in particular—in critically ill patients [2, 7]. We previously described the development of a novel multiplex real-time PCR assay to detect microbial DNA directly in whole blood (which will further be referred to as BSI-PCR) [8]. This test combines 17 individual PCRs, creating a broad panel of species- and genus-specific DNA targets as well as some generic ones [8]. We previously compared BSI-PCR with standard blood culture (BC) in 347 samples and found that sensitivity was acceptable for most bacterial species-specific PCRs (varying from 65 to 100%), yet still remained insufficient for yeasts and some generic targets [8]. However, that study could not assess the clinical significance of positive BSI-PCR findings, due to a design that focused on blood samples that were merely selected based on the result of a paired BC, which itself has imperfect sensitivity.

In contrast, the current study was performed in a clinically relevant population of patients with suspected sepsis at ICU admission. Moreover, we did not solely compare the new test to BC results but used a prospectively recorded clinical reference diagnosis which considered all cultured pathogens that could potentially be relevant in relation to the presumed site of infection. However, this implies that, for negative BSI-PCR results, no certain distinction could be made between false negative findings (i.e., the test potentially failed to detect a clinically relevant pathogen) and true negative findings (i.e., the non-detected microorganism was merely a colonizer or contaminant). Therefore, our study aimed at estimating the positive predictive value (PPV) and the false positive proportion (FPP) of BSI-PCR in a clinically relevant context, without specifically evaluating other measures of diagnostic accuracy.

## Materials and methods

### Study population

Patients were prospectively enrolled as part of the Molecular Diagnosis and Risk Stratification of Sepsis (MARS) cohort in two tertiary ICUs in the Netherlands between January 2012 and June 2014. Ethical approval for the study was provided by the Medical Ethics Committee of the University Medical Center Utrecht, including an opt-out consent method (IRB No. 10-056C). We selected consecutive patients with presumed sepsis, who had been admitted to ICU within 48 h of infection onset (i.e., start of antimicrobial treatment) and in whom blood cultures were obtained at the discretion of the attending physician. Enrolment took place before the introduction of the sepsis-3 criteria [9]; thus, patients were included based on the sepsis-2 “severe sepsis” and “septic shock” definitions [10]. However, only six (2%) subjects did not fulfil current sepsis-3 criteria in retrospect. Patients already receiving treatment for another infection for more than 2 days were excluded.

### Sample collection and BSI-PCR assay

BSI-PCR samples were collected at the same time that BCs were performed during ICU admission. For each set of BCs (i.e., a single set of aerobic and anaerobic vials), a 5 mL blood tube was drawn from the same catheter hub or venepuncture site. For patients having multiple or subsequent samples, we analyzed only the first available one. Blood samples were stored at 4 °C for a maximum of 3 days before processing. BSI-PCR was performed in 5 mL whole blood, as described in detail previously, resulting in DNA isolate volumes representative of 0.71 mL blood per individual PCR [8]. Technicians performing tests and interpreting PCR results were blinded for clinical and microbiological findings. Of note, the multiplex BSI-PCR panel consisted of 17 individual PCRs; thus, each sample could yield multiple positive results.

### Reference diagnosis

For each suspected sepsis episode, both the post hoc plausibility of true infection and the most likely causative pathogen(s) were prospectively adjudicated by trained physicians, who attended daily multidisciplinary clinical meetings and had full access to all clinical data as part of the MARS study [11]. For use as reference diagnosis, infection was considered confirmed only if the plausibility of infection had been rated either probable or definite according to criteria described previously [11]. Reference pathogen(s) were those that had been assigned as likely causative microorganism(s) during prospective adjudication considering all microbiological evidence available (i.e., acquired either before, during, or after ICU admission). However, it should be noted that the reference pathogen was classified as “unknown” if no pathogens had been identified (ever), and if all cultured microorganisms were considered to be not potentially relevant in relation to the presumed site of infection. All observers contributing to the reference diagnosis were blinded for BSI-PCR results.

Of importance, no distinction could be made between false negative and true negative BSI-PCR findings, because the reference pathogens incorporated all identified pathogens in relation to the presumed site of infection without a certain distinction between true causative pathogens and colonization. BSI-PCR should thus not be expected to identify all potential pathogens considered by the reference diagnosis. Consequently, not all microorganisms “missed” by BSI-PCR were classified as a false negative result. For example, for a patient with hospital-acquired pneumonia in whom sputum cultures yielded growth of both *Staphylococcus aureus* and *Pseudomonas aeruginosa*, both species were registered as a potential reference pathogen. However, we did not consider BSI-PCR to return a false negative result if the test identified only one of these two bacteria. For this reason, our study focused on the interpretation of positive BSI-PCR results and does not include evaluation of pathogens potentially “missed” by BSI-PCR.

### Evaluation of BSI-PCR

In our primary analysis, we compared BSI-PCR results to the reference pathogen(s) and classified all discordant PCR findings as false positive. Since BSI-PCR yields 17 results per patient, the test could simultaneously have both true and false positive findings. Such instances were classified as “partial true positive.” In a secondary analysis, we compared BSI-PCR results with the results of BC. Furthermore, we assessed whether cycle times (Ct)–values differed between true and false positive PCR results.

In a pre-planned discrepancy analysis, we reassessed all positive BSI-PCR results that were discordant to the reference (including “unknown” reference pathogens) in order to adjust for potentially suboptimal sensitivity of the culture-based techniques used in establishing the reference diagnosis. Case vignettes (see Appendix I) were created and evaluated by an expert panel consisting of intensivists (LD or OC) and clinical microbiologists (DO or MB). All detections by BSI-PCR that were not included as reference pathogens were subsequently re-classified as true positive, false positive, or undetermined. Of note, panelists had agreed that bacterial translocation was to be considered a plausible cause of DNAemia by *Enterobacteriaceae*, Enterococci, and *Candida* in sepsis patients having intestinal ischemia, bowel perforation, or overt abdominal compartment syndrome, and in the presence of advanced liver failure [12, 13].

In a final explorative analysis, we assessed the possible impact of BSI-PCR on the choice of antimicrobial therapy as given on the first day in ICU. As the sensitivity of BSI-PCR is currently considered insufficient to rule out bacteremia in critically ill patients with confidence [8], we did not evaluate whether de-escalation was indicated based on BSI-PCR results. A clinical microbiologist (DO or MB) thus evaluated only whether positive PCR results would have led to a broader choice in antimicrobial coverage.

### Statistical analysis

For the primary analysis, we calculated PPV and FPP with exact confidence intervals (CI). FPP was calculated by dividing the number of false positive results by the total number of individual PCR’s performed. Since no certain distinction could be made between true negative and false negative BSI-PCR results (as explained above), it was unfeasible to calculate estimates for sensitivity, specificity, and negative predictive values. For the discrepancy analysis, we adjusted these parameters based on the expert panel adjudication. Since this reclassification introduced an “undetermined” category, a range of estimates was calculated (i.e., first classifying the undetermined cases as false positives, and subsequently as true positives). Differences between groups were assessed using chi-square, Wilcoxon rank sum, or Fisher’s exact tests, as appropriate. All analyses were performed using SAS Enterprise Guide 7.1 (SAS Institute, Cary, NC), and figures were made using GraphPad Prism version 7.04 (GraphPad Software, La Jolla, CA, USA).

## Results

### Patient and infection characteristics

**Fig. 1:**
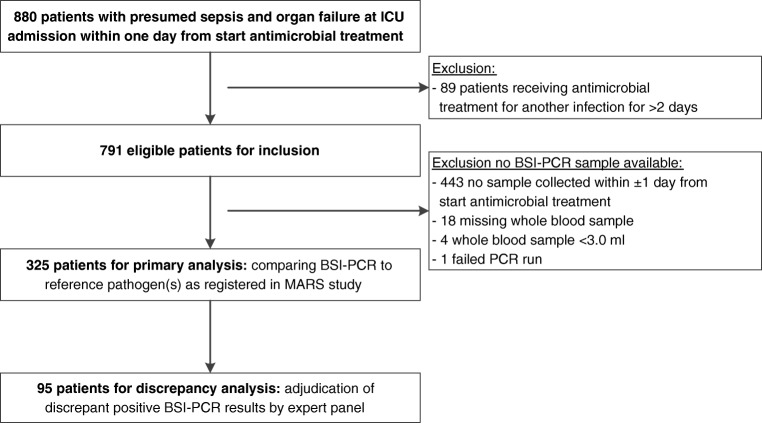
Patient inclusion. *BSI* bloodstream infection; *ICU* intensive care unit; *PCR* polymerase chain reaction

Among 791 eligible patients admitted to ICU with presumed sepsis, 325 (41%) were included in the current study (Fig. 1). Study exclusions were mostly due to omissions of BC and/or paired BSI-PCR blood sampling at the time of ICU admission. Of note, many patients (also) had cultures taken prior to ICU presentation and all subjects were clinically suspected of (and treated for) sepsis. However, based on post hoc adjudication, infection likelihood was categorized as confirmed, uncertain, and ruled out in 210 (65%), 88 (27%), and 27 (8%) patients, respectively (Table 1). Among study patients, median APACHE-IV scores were 85 (interquartile range (IQR) 68–109) and half of patients presented with septic shock. Sepsis had community-acquired onset in 188 (58%) patients, and the most common infection sites included the respiratory tract and abdomen.

**Table 1:** Characteristics of 325 patients with presumed sepsis at ICU admission

Variables	Study population (*n* = 325)
Patient characteristics	
Sex (male)	186 (57%)
Age (years)	63 (52–72)
Chronic obstructive pulmonary disease	45 (14%)
Diabetes mellitus	65 (20%)
Solid or hematologic malignancy	84 (26%)
Chronic renal insufficiency	46 (14%)
Charlson comorbidity index	1 (0–2)
Immune deficiency	57 (18%)
Admission characteristics	
Recent surgery	62 (19%)
Prior ICU admission	55 (17%)
APACHE-IV score	85 (68–109)
C-reactive protein (mg/L)^a^	212 (95–305)
White blood cell count (10^9^/L)^a^	16 (10–21)
Septic shock	164 (50%)
SOFA-score on day 1	9 (7–11)
Length of stay in ICU	5 (3–13)
Mortality after 30 days	107 (33%)
Infection characteristics	
Community-acquired onset	188 (58%)
Presumed source:	
- Lower respiratory tract	152 (47%)
- Abdomen	79 (24%)
- Urinary tract	24 (7%)
- Other	61 (19%)
- Unknown	9 (3%)
Post hoc plausibility of infection:^b^	
- Definite	127 (39%)
- Probable	83 (26%)
- Uncertain	88 (27%)
- Ruled out	27 (8%)

*APACHE* Acute Physiology And Chronic Health Evaluation, *ICU* intensive care unit, *LOS* length of stay, *SOFA* Sequential Organ Failure Assessment. Data are presented as frequencies (%) or medians (Q1–Q3)

^a^Missing data: C-reactive protein *n* = 106 (33%), and white blood cell count *n* = 3 (2%)

^b^Based on post hoc assessment of plausibility of infection by trained physicians based on all clinical information as described elsewhere [11]

### BSI-PCR findings

**Fig. 2:**
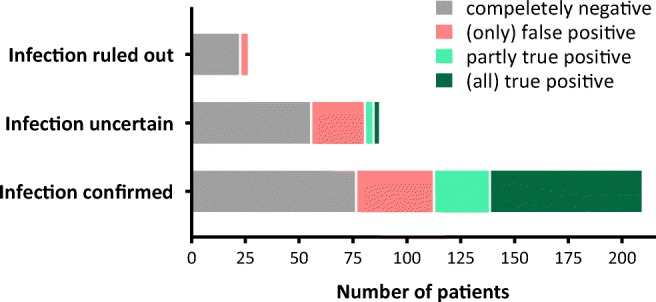
BSI-PCR performance on patient level by infection plausibility of reference diagnosis. Plausibility of infection was considered confirmed in patient with a post hoc likelihood of infection rated as probable or definite. Since BSI-PCR is a multiplex real-time PCR containing 17 microbiological targets, multiple positive results were possible per patient. Therefore, patients with both true positive and false positive results were classified as “partly true positive”

BSI-PCR yielded one or more pathogen detections in 169 (52%) patients, with a median of 2 (IQR 1–2) positive PCRs per patient. According to the primary analysis, these results were considered true positive in 74 (44%), partial true positive in 30 (18%), and false positive in 65 (38%) patients (Fig. 2). Among these latter 65 subjects, infection was considered ruled out in four and the causative pathogen was unknown in 30 according to the reference diagnosis. By comparison, 47 pathogens had been identified by BC in 43 (13%) patients and seven of these isolates had been considered contaminants during the prospective adjudication process. BSI-PCR identified 25 (63%) of the remaining 40 BC isolates.

In the discrepancy analysis, the expert panel adjudicated 128 apparently false positive PCR results in 95 patients (i.e., 35 detections in 30 patients classified as partial true positive, and 93 detections in 65 patients classified as false positive). Thirty-four (27%) of these PCR results were unanimously reclassified as true positive, whereas 10 (8%) and 84 (66%) discordant results remained unresolved and false positive, respectively. Among the 169 patients having (one or more) positive BSI-PCR results, panel review thus resulted in adjusted true positive, partial true positive, and false positive rates of 100 (59%), 6 (4%), and 63 (37%), respectively.

### Assessment of individual PCR performance

Among 325 patients, multiplex BSI-PCR generated a total of 5525 single PCR results, of which 295 were positive. This resulted in a PPV of 167/295 (57%, 95% CI 51–62%) overall, with estimates for individual species-specific and generic PCRs ranging 16–82% and 46–91%, respectively (Table 2). Overall FPP was 2% (95% CI 2–3%) based on the 128 discordant positive results observed according to the primary analysis (individual estimates ranging 0–8% and 0–4%, respectively). Ct-values were significantly lower for the 167 true positive detections than for the 128 false positive results (median 31.9 (IQR 27.8–35.7) versus median 37.9 (IQR 35.6–40.0); *p* < 0.001).

**Table 2:** Evaluation of positive BSI-PCR results in 196 critically ill patients with presumed sepsis

Pathogen or target		Primary analysis^a^				Discrepancy analysis^b^			
		True positive		False positive		True positive range		False positive range	
	(*n*)	*n* (PPV)	(95% CI)	*n* (FPP)	(95% CI)	*n*	(PPV)	*n*	(FPP)
A. Species-specific PCRs									
*E. faecalis*	(16)	7 (44%)	(20–70)	9 (3%)	(1–5)	11–12	(69–75%)	4–5	(1–2%)
*E. faecium*	(11)	9 (82%)	(48–98)	2 (1%)	(0–2)	10	(91%)	1	(0%)
*S. aureus*	(34)	15 (44%)	(27–62)	19 (6%)	(4–9)	15–18	(44–53%)	16–19	(5–6%)
*S. pneumoniae*	(17)	12 (71%)	(44–90)	5 (2%)	(1–4)	13	(76%)	4	(1%)
*A. baumannii*	(1)	0	–	1 (0%)	(0–2)	0–1	(0–100%)	0–1	(0–0%)
*E. coli*	(58)	33 (57%)	(43–70)	25 (8%)	(5–11)	45–46	(78–79%)	12–13	(4–4%)
*Klebsiella*	(11)	7 (64%)	(31–90)	4 (1%)	(0–3)	9–10	(82–91%)	1–2	(0–1%)
*P. aeruginosa*	(32)	5 (16%)	(5–33)	27 (8%)	(6–12)	5–7	(16–22%)	25–27	(8–8%)
*C. albicans*	(0)	–		–		–		–	
*C. glabrata*	(0)	–		–		–		–	
*C. krusei*	(0)	–		–		–		–	
Total A.	(180)	88 (49%)	(41–56%)	92 (3%)	(2–3%)	108–117	(60–65%)	63–72	(2–2%)
B. Generic PCRs									
*Enterococcus*	(25)	12 (48%)	(28–69)	13 (4%)	(2–7)	16–17	(64–68%)	8–9	(2–3%)
*Staphylococcus*	(24)	11 (46%)	(26–67)	13 (4%)	(2–7)	12	(50%)	12	(4%)
Gram-positive	(30)	25 (83%)	(65–94)	5 (2%)	(1–4)	30	(100%)	0	–
Gram-negative	(33)	30 (91%)	(76–98)	3 (1%)	(0–3)	33	(100%)	0	–
3*Candida*	(2)	1 (50%)	(1–99)	1 (0%)	(0–2)	2	(100%)	0	–
Pan-*Aspergillus*	(1)	0	–	1 (0%)	(0–2)	0	–	1	(0%)
Total B.	(115)	79 (69%)	(59–77)	36 (2%)	(1–3)	93–94	(81–82%)	21–22	(1–1%)
Total	(295)	167 (57%)	(51–62)	128 (2%)	(2–3)	201–211	(68–72%)	84–94	(2–2%)

*n*, number of positive BSI-PCR results (multiple positive results possible per patient); *CI*, confidence interval; *PPV*, positive predictive value (= true positive/(true positive + false positive)); *FPP*, false positive proportion (= false positive/total number performed (*n* = 325 per PCR))

^a^For the primary analysis positive BSI-PCR results were compared with the reference pathogens based on a prospective registration of most likely causative pathogen(s) within the MARS study

^b^For the discrepancy analysis, false positive BSI-PCR results based on the primary analysis were adjudicated by an expert panel. Since 10 results were classified as “undetermined” by the panelists, a range was calculated for the PPV and FPP by classifying the undetermined results firstly as false positive and subsequently as true positive

Adjudication of discordant positive results by the expert panel resulted in an adjusted overall PPV ranging from 68 to 72%, depending on whether results classified as undetermined were analyzed as false or true positive finding (Table 2). Overall FPP remained 2%. We observed no positive methicillin resistance PCRs, none of the species-specific Candida PCRs were positive, and the PCRs for the extended spectrum beta-lactamase resistance gene (CTX-M1,9) were uniformly negative as well; this precluded calculation of PPV and FPP for these targets.

### Potential therapeutic impact of BSI-PCR findings

Among the 169 patients with positive BSI-PCR findings, there were 55 (33%) cases in which the identified pathogens were not covered by the antimicrobial regimen as given on the first day in ICU. Based on expert review, BSI-PCR results could have led attending physicians to consider broadening of initial antimicrobial coverage in 37 (22%) of these episodes, mainly to assure additional treatment of *P. aeruginosa* (*n* = 25) and Enterococci (*n* = 6). However, as findings were considered false positive in 20 (54%) of these 37 patients, the potential benefit of BSI-PCR-guided antibiotic therapy was limited to 17 (5%) of 325 patients at most.

### Patients with negative BSI-PCR results

Among 156 (48%) patients with negative BSI-PCR, infection was classified as ruled out according to the reference diagnosis in 23 (15%) cases. Six (4%) other patients had only viral pathogens and could therefore also be considered to have a true negative BSI-PCR result. Among the 127 remaining patients, infection was considered uncertain and confirmed in 54 (35%) and 73 (57%) cases, respectively. Particularly in the latter group, BSI-PCR probably represented a false negative result. Among these were five patients in whom BC grew *Enterobacteriaceae*, *Bacteroides* species, or Gram-positive pathogens.

## Discussion

In this diagnostic cohort study, we evaluated a novel multiplex PCR test on whole blood for rapid pathogen detection in ICU patients with presumed sepsis. BSI-PCR detected one or more pathogens in half of the patients. These findings were considered true positive in 44% and partial true positive in 18% of the episodes, when compared with prospectively assigned reference pathogens. PPV was 68% after adjudication of discrepant pathogens detected by BSI-PCR. False positive results were observed in about a third of included patients, and BSI-PCR failed to identify a reference pathogen in 54% of all patients in whom infection was considered present.

This study demonstrates that PCR-based technologies may complement BC in pathogen identification in sepsis patients, because of additionally identified causative pathogens and theoretically faster results. This is in line with previously performed evaluations of other molecular-based assays for pathogen identification directly on whole blood [5, 14–19]. In general, these validation studies show relatively low sensitivity and reasonable specificity for the multiplex-PCR assays but results are very heterogeneous [2, 5]. Furthermore, direct comparison of the PPV of BSI-PCR observed in our study with other results is hampered by large differences in study populations and used reference diagnoses.

Based on the current study of BSI-PCR performance, as well as a previously published direct comparison with paired BC samples obtained from a different patient population [8], we hold the opinion that the diagnostic accuracy of the test is still insufficient for implementation in clinical practice at this time. The first issue relates to suboptimal sensitivity. In fact, BSI-PCR did not (correctly) identify a pathogen in 54% of patients with a confirmed infection. Of note, this false negative rate was lower than that of the paired BC, which did not yield growth in 82% of these cases. Furthermore, dissemination of (non-viable) pathogens to the bloodstream is required for a positive BSI-PCR but it is unclear to what extent this occurs in patients with various local sites of infection. Moreover, BSI-PCR is also not designed to fully replace culture-based methods, since the assay provides only limited information about antimicrobial susceptibility and does not cover infrequently found bacteria and fungi. A second issue relates to the frequent occurrence of false positive detections. Although the FPP of individual PCRs was mostly acceptable, the multiplex nature of BSI-PCR yields a large number of test results, thus increasing the overall rate of false positive findings to 29%. These may be due to DNA contamination during sample processing in the laboratory or by the presence of (non-viable) microbes in the circulation. In clinical practice, it would be challenging to distinguish true from false positive results but the semi-quantitative measurement of microbial DNA load (expressed as Ct-values) appeared to be discriminative and could be used as guidance. In the current study, we did not recommend specific cut-off points for Ct values, as optimal trade-off between sensitivity and specificity will likely vary across clinical scenarios.

An important strength of this study was the prospective registration of reference diagnosis and reference pathogens by trained observers within the parent cohort of the MARS study. Furthermore, we corrected our estimates of test accuracy for the suboptimal sensitivity of conventional cultures through re-adjudication by an expert panel. Finally, we included a large population of presumed sepsis patients with various underlying infectious diseases, which created a heterogeneous population representative for clinical practice. Nevertheless, we also consider the reference diagnosis as suboptimal, because, based on culture results, no confident distinction could be made between colonization and true causative pathogens. In addition, inter-observer agreement for the registration of causative pathogens was previously reported to be only 70% [11], and because classification was based on clinically performed microbiological testing, which may still be incomplete. Therefore, future studies should assess the diagnostic accuracy of BSI-PCR in a prospective intervention study, which should incorporate the assessment of test results on antimicrobial therapy and cost effectiveness.

In conclusion, this clinical evaluation demonstrated that BSI-PCR had only moderate PPV. Furthermore, the test identified potential pathogen(s) in no more than half of patients having a high likelihood of infection. This precludes any consideration of clinical implementation of BSI-PCR at this time. However, the assay is still under development and its accuracy will likely be improved by the use of larger blood input volumes and implementation on a fully automated cartridge-based platform for sample processing. In the future, the potential to provide rapid results could make BSI-PCR—and similar assays—of additional value for pathogen detection in critically ill patients with presumed sepsis.

## Electronic supplementary material

ESM 1 (PDF 351 kb)
