# Supplementary material for: Moderate positive predictive value of a multiplex real-time PCR on whole blood for pathogen detection in critically ill patients with sepsis
Source: Eur J Clin Microbiol Infect Dis. 2019 Jun 26;38(10):1829–36. doi: 10.1007/s10096-019-03616-w (PMC6778535; doi:10.1007/s10096-019-03616-w)
Supplement: Supplementary file 1 — (PDF 351 kb) [file 10096_2019_3616_MOESM1_ESM.pdf]

ELECTRONIC SUPPLEMENTARY MATERIAL

Moderate positive predictive value of a multiplex real-time PCR on whole blood for pathogen detection in critically ill patients with sepsis

Kirsten van de Groep, Martine P. Bos, Meri R.J. Varkila, Paul H.M. Savelkoul, David S.Y. Ong, Lennie P.G. Derde, Nicole P. Juffermans, Tom van der Poll, Marc J.M. Bonten, Olaf L. Cremer, *on behalf of the MARS consortium.*

Content:

Appendix I. Template of case vignette used for expert panel

APPENDIX I. Template of case vignette used for expert panel

**CASE VIGNETTE CLINICAL VALIDATION BSI-PCR**<br>**Initials reviewer:**			
**Patient_TK**		**Date sepsis**	
**Hospital admission date**<br>**ICU admission date**		**Date PCR sample**	

Clinical summary:

X-year-old female. Hospital admission on DATE, because of an ischemic CVA. Recovery complicated by two times pneumosepsis with two consequent ICU-admissions. During first ICU admission *E. coli* in sputum culture and treatment with cefotaxime. DATE again fever, sputum, leucocytes 19, sputum culture positive with *S. aureus*. On DATE re-admission to the ICU with pneumosepsis and septic shock, antimicrobial treatment initiated. Differential diagnosis re-activation of Crohn disease, but ruled out on CT-abdomen (signs of stenosis, but no evident new pathology).

Relevant medical history:
Chrohn disease.

Antimicrobial treatment:
DATE: empiric metronidazol/vancomycin/gentamycin
DATE: de-escalation based on culture results to flucloxacillin

Relevant culture results

SC 23/1	E. coli
SC 28/3	S. aureus (+++)
SC 2/3	S. aureus (++) ; C. krusei ; throatflora
UC 2/3	C. krusei
BC 2/3	S. epidermidis (1 out of 2 sets)
BC 1/3 & 28/2	Negative

MARS classification

Primary Site	HAP (probable)	Secondary Site	Sec. peritonitis (none)
Pathogens:	S. aureus		-

**PCR-results**			REVIEWER:
P. aeruginosa	(Ct 40)	**Classification PCR-result (TP/FP/?)**	
S. pneumoniae	(Ct 40)	**Classification PCR-result (TP/FP/?)**	

Short comment reviewer:
